# The treatment of persistent spinal pain syndrome with epidural pulsed radiofrequency: improvement of the technique

**DOI:** 10.3389/fneur.2023.1236270

**Published:** 2023-10-16

**Authors:** Alessandro Dario, Sergio Capelli

**Affiliations:** Neurosurgical Clinic of Insubria University, ASST Settelaghi, Varese, Italy

**Keywords:** pulsed radiofrequency, persistent spinal pain syndrome, pain, epidural, electrocatheter

## Abstract

**Background:**

In Persistent Spinal Pain Syndrome (PSPS), Pulsed Radiofrequency (PRF) is a used procedure. The results of PRF in PSPS performed with an electrode placed through the sacral hiatus were reported to be successful on pain in only 32% of patients. We have tried to improve the results by applying a new system to PRF.

**Methods:**

Ten patients were treated with PRF application through a steerable epidural catheter with a reference electrode outside the foramen. This method was named “Optimization Current Flow” (OCF). The duty cycle of PRF was 2 × 10 msec and total exposure time was 150 s. Follow up was planned for 1, 3, and 6 months. The NRS score was considered to be the primary outcome.

**Results:**

In the first 10 patients treatment was successful (69% of the patients) at 6 months follow-up.

**Conclusion:**

This new modality of PRF in patients with PSPS seems to be superior to procedures done with a needle. Further prospective double-blind randomized long-term studies with a significant number of patients are required to validate this technique as there is a need to improve PRF results in PSPS.

## Introduction

Failed Back Surgery Syndrome (FBSS) is a distinct medical condition that affects 15% to 30% of patients undergoing lumbar spine surgery (1, 2). Recently this conditions has been has been renamed Persistent Spinal Pain Syndrome (PSPS) type 2 (3) and is marked by radicular neuropathic leg pain sometimes combined with low back pain.

Scar tissue is formed following surgery, involving the dorsal root ganglion and nerve roots, but epidural fibrosis is considered to be a radiological entity not correlating with the patient’s complaints (4) and the degree of fibrosis is equal in symptomatic and asymptomatic patients (5).

Treatment varies from psychological support on one side of the scale to re-operation on the other. Minimally invasive procedures such as spinal steroids injections, lysis of adhesions, epiduroscopy and both pulsed and continuous radiofrequency have a certain popularity but the results can be modest and sometimes temporary (6, 7).

Pulsed Radiofrequency (PRF) close to the dorsal root ganglion is one of the therapeutic options, used mainly in patients with limb pain. The analgesic effect of PRF is not related to permanent physical neural damage (8) as in radiofrequency thermocoagulation (9).

The mechanism of action could be a neuromodulatory-type process, which alters the synaptic transmission or the excitability of C-fibers (10). Although the PRF could be applied to different site of the afferent nervous pathways (10), we believe that the ganglion is the best target in cases of PSPS type 2 that cause pain in the entire lower limb.

Two approaches are possible. An electrode may be inserted by the percutaneous transforaminal route, or a catheter may be used, entering through the sacral hiatus in the epidural space. Both methods have an acceptable rate of success in non-operated patients (11, 12) and both have disappointing results in patients with PSPS type 2 (7, 13). Literature data on this issue are scarce and contain data on small numbers of patients (13–17). The difference with the best results in non-operated patients could be due to technical problems, such as the presence of dense scar tissue in the spinal canal or bone grafts blocking the way to optimal electrode positions. Another option is partial blocking of the current by scar tissue around the ganglion, but this is less likely because at the usual RF frequency of 400 KHz the current easily penetrates such barriers. The aim of this study is to propose an improvement of the PRF technique to obtain better clinical results in pain control evaluated by the Numerical Rate Scale (NRS) score.

## Current PRF technique and technical improvements in the new technique

There are currently few studies on the use of PRF in patients with PSPS type 2, and almost always the series almost always include several etiologies other than PSPS type 2. Most of these reports use needle electrodes for the transforaminal route (13, 18–20). Among the patients, >50% pain relief was found at follow-up in 45% (16), 40% (13), and 33% (19).

Three of these studies reported few cases of PSPS type 2 (15, 17, 20). The use of an epidural catheter has been described in three studies (12, 21, 22). In a clinical series of only PSPS type 2 long-term (from 3 to 6 months follow-up) pain success varied from 32% (21) to 48% (12) of a series with pain caused by various pathologies; in the patients of Gulduren Aydin et al. (22) the technique proved to be statistically effective both in patients with PSPS type 2 and in other diseases. In all these studies the cathode was the skin plate and the anode the cannula with active tip electrode or the epidural catheter tip.

In three studies (23–25) patients with lumbosacral radicular pain were subjected to PRF using two catheter needles with active tips less than 1 cm apart. This technique was proposed to improve clinical results and this objective was achieved in the randomized controlled trial (23). However no patients had PSPS type 2, which was indeed an exclusion criterion.

Compared with monopolar PRF, it has been suggested that bipolar PRF would produce a denser and larger electrical field (23, 24) with better results on lumbosacral radiculopathy (24). Whith a monopolar current output the covered area is 12.8 mm × 7.8 mm, while the covered area of the bipolar PRF should be 15.5 mm × 11.8 mm thus bipolar PRF can cover the Dorsal Root Ganglion (DRG) more sufficiently. However with this technique the area of administration of the current does not cover the entire ganglion unlike the new technique we describe below which for anatomical and current administration reasons covers the entire ganglion.

The setting of the treatments in the various series is usually 120 s with 45 volts at temperature of 42° using the cannula needle, while in cases treated with the epidural electrode (12, 21, 22) the voltage varies from 65 to 80 volts for 240 s. The described follow-up varies from 1 month to 3 years (13, 17–19).

**Figure 1:**
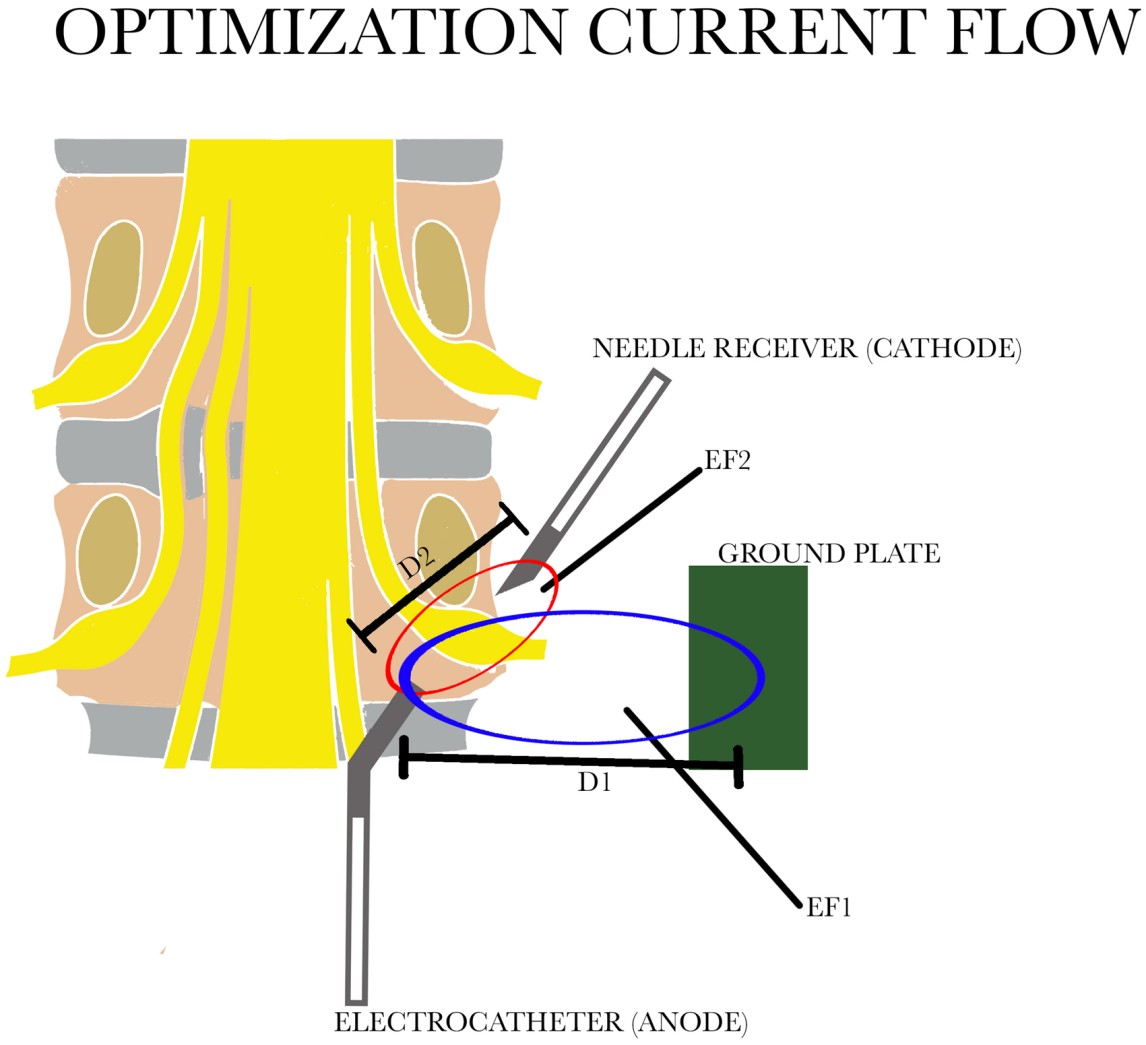
Comparison of the two electric fields: in blue EF1 with the distance between the electrocatheter and the ground plate D1; in red EF 2 with the distance between the electrocatheter and the needle receiver D2. D1 is greater than D2. EF, electric field.

When the conventional monopolar technique is used such fields are found in a spherical zone around the active tip of the electrode, and a distance of approximately 2–3 cm is reached when 45 V are applied. Control of this distribution of electricity on the DRG is not always possible: in fact the distance D1 is not sufficient to cover the area of the entire ganglion (Figure 1).

We have tried to improve this procedure by using bipolar application of PRF with a relatively large distance between the electrodes. This changes the geometry of the zone where the relevant fields are found into a prolate spheroid with its long axis along the line that connects the electrodes (Figure 1). Contrary to the situation during monopolar application, this zone (distance D2) can now be controlled by adjusting the voltage, the distance between electrodes and the position of the reference electrode. The new configuration therefore provides a more efficient distribution of the relevant electric fields. We have named this system “Optimization Current Flow” (OCF). A 16 G Tuohy needle was placed, under fluoroscopy, through the hiatus sacralis into the sacral epidural space, up to a level just caudal to the foramen of S3.

**Figure 2:**
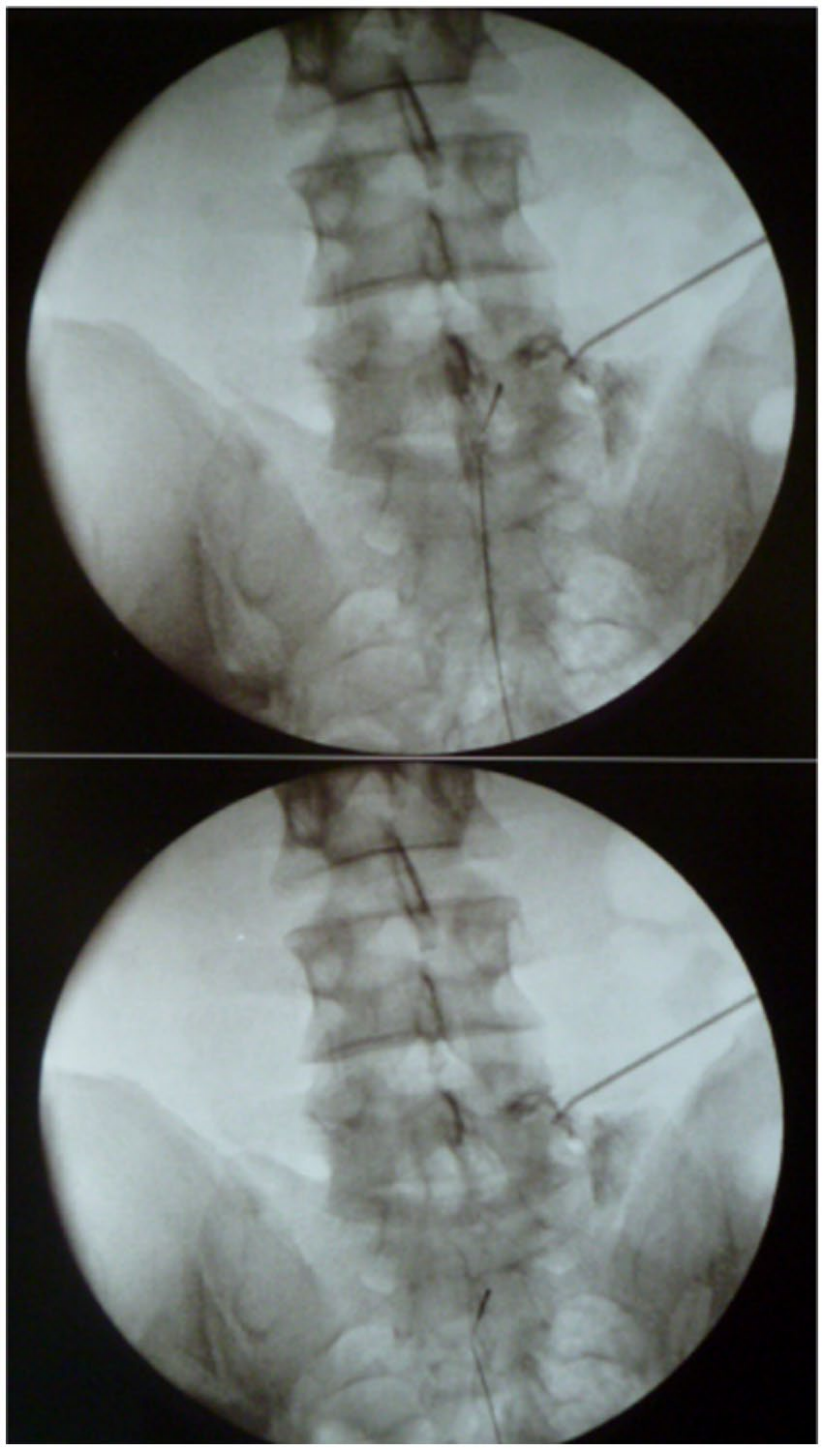
Radicolography of L5 and S1 with Optimization Current Flow (OCF).

A 4 F diameter microelectrocatheter type RC-Cath (ACACIA^®^, Milan, Italy) with a 10 mm steerable tip, was then introduced and directed towards the relevant ganglion recess. An 18 G, 100 mm needle with an atraumatic active tip 10 mm long, was placed close to the foramen from a posterolateral approach (Figure 2), acting as a reference electrode (cathode).

Before the start of the procedure, epidurography was performed with an equal mixture of water soluble contrast medium (Jopamiro 300 mg/mL) and normal saline solution to rule out incorrect placement of the catheter. Sensory stimulation at 50 Hz was performed at each level to check the correct positioning. PRF was then applied at 2 Hz × 10 ms and 25 V for a total exposure time of 150 s for every level treated. The average impedance was 275.8 ohms (190–350 ohms). We used 150 s because the coverage area of the ganglion is total. For the same reason it is pointless to use electrodes with a 15-mm tip.

Inclusion criteria were persistent lower back pain and radiating pain of the leg with an NRS-score of at least 5 on a 0–10 scale, and duration of at least 6 months, following single or multiple surgical interventions on the lumbar spine. At clinical examination none of the patients had evidence of neurological deficit. The first 10 patients were considered: eight had lumbar surgery for removal of a lumbar discal hernia at a single level (two patients at two levels), and two patients for lumbar stenosis had bilateral pain.

The NRS score was used to monitor the clinical effect and was considered as the primary outcome. Success was defined as a fall of 3 points or more. All demographic data are described in Table 1. Statistical analysis was done using the one way ANOVA test for paired data,^1^ and *p* < 0.05 was considered to be significant.

**Table 1:** Patients’ demographic data.

Patient	Age	Sex	Etiology & spinal level	Duration and characteristics of pain	PRF Level & side	Preoperative NRS score	NRS at 1 month FU	NRS at 3 months FU	NRS at 6 months FU
1	49 years	Male	Disc herniation L5-S1 L	12 months left sciatica	L5-S1 L	9	2	3	3
2	27 years	Female	Disc herniation L4-L5 L	8 months left sciatica	L4-L5 L	10	3	4	8
3	51 years	Female	Disc herniation L3-L4 L	7 months L lumbosciatica	L3-L4 L	8	3	3	3
4	65 years	Male	Lumbar stenosis L3-L4 Bil	24 months bil lumbosciatica	L3-L4 bil	8	2	2	2
5	53 years	Male	Disc herniation L4-L5 R	13 months R lumbosciatica	L4-L5 R	7	2	3	5
6	57 years	Female	Disc herniationL4-L5 & L5-S1 R	47 months R sciatica	L4-L5 R	7	3	3	7
7	59 years	Male	Disc herniation L4-L5 L	15 months L lumbosciatica	L4-L5 L	8	3	3	3
8	45 years	Female	Disc herniation L4-L5 & L5-S1 R	8 months R lumbosciatica	L4-L5 R	7	3	2	2
9	71 years	Female	Lumbar stenosis L3-L4 bil	20 months bil lumbosciatica	L3-L4 bil	8	3	3	3
10	59 years	Male	Disc herniation L5-S1 R	16 months R lumbosciatica	L5-S1 R	8	2	2	3

Patients’ demographic data; L, left; R, right; bil, bilateral; PRF, pulsed radiofrequency; NRS, numerical rate scale; FU, follow up.

The Medical Management Direction of ASST Settelaghi (Varese, Italy) hospitals requested only an informed consent read and signed by each patient as this was a no-experimental procedure with EEC marked material, since the applied medical treatment did not exceed medical standards.

## Results

The PRF was administered bilaterally at the L3-L4 level in patients with bilateral pain, and in other cases at the level of the previous lumbar operation.

**Figure 3:**
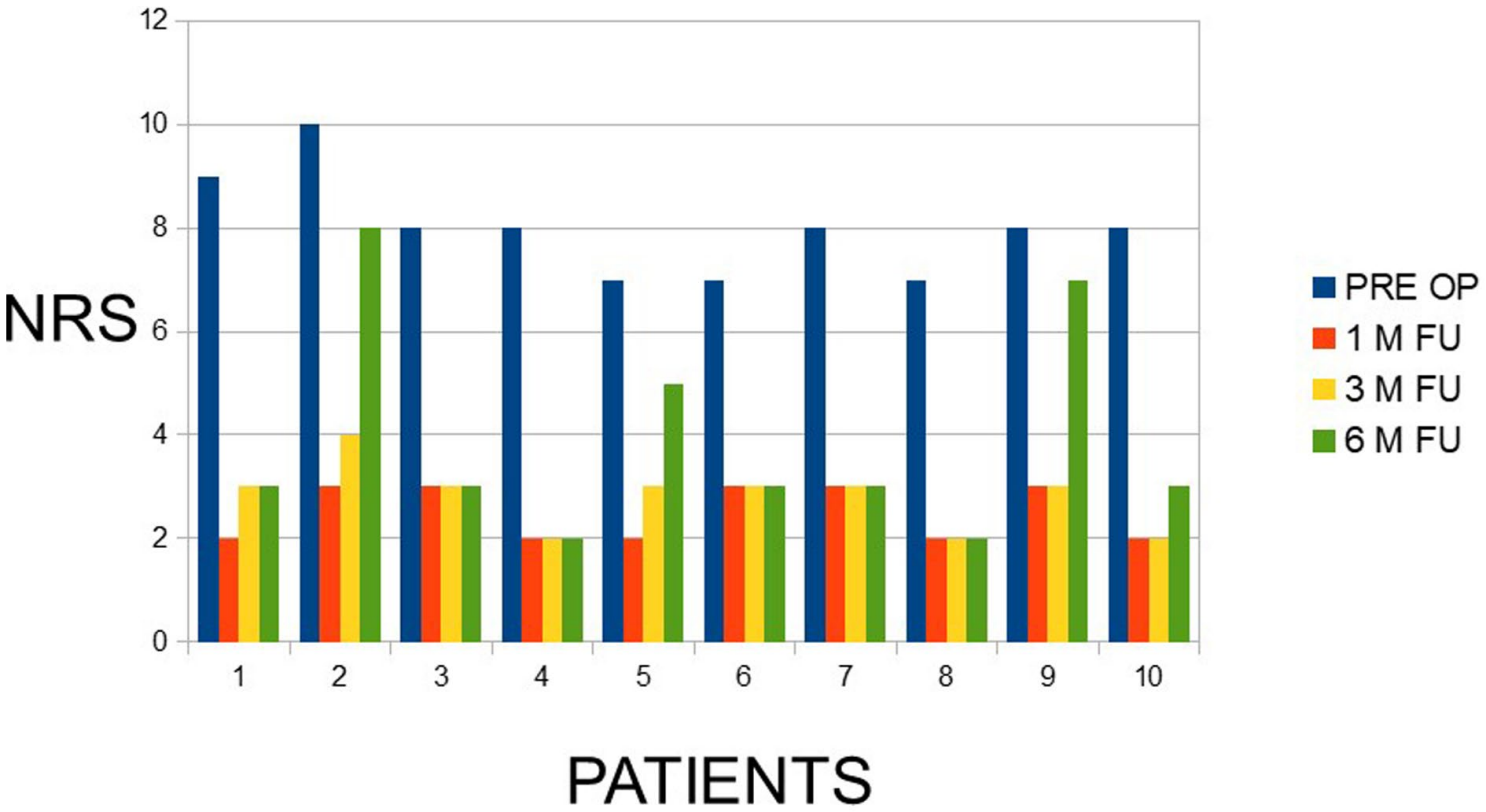
NRS scores, preoperative and at programmed follow-up.

The treatment was successful at 1, 3 and 6 months’ follow-up in 69% of the patients. The improvement from preoperative median score of 8 the score improved to 3.9. There were no complications. The ANOVA analysis (Figure 3) showed a highly significant change from the initial level of the NRS score to all follow up values (*p* < 0.0001), demonstrating that the goal of reducing pain was achieved with statistical significance during follow-up.

## Discussion

Currently the treatment of drug-resistant PSPS type 2 in patients with radicular pain is based on epidural injections, percutaneous adhesiolysis and spinal cord stimulation (26); however the second option requires a longer surgical time with the risk of several reported adverse effects seen in both percutaneous and epidural adhesiolsis including infection, Infection, postoperative weakness, sensory deficit, rash, weight gain, head and neck pain, wound pain, sciatic pain, low back pain, dural tear, bleeding, blindness, and apnea (27).

The Spinal Cord Stimulation (SCS) requires the implantation of a prosthesis with the related problems. Ablative radiofrequency used for somatic lower back pain is a destrutive procedure that cannot be used at the gangliar or epidural level in PSPS type 2. Pulsed radiofrequency does not require implants and no major complications are reported (22): however it is necessary to improve the clinical results.

The first results of our study have to be interpreted with caution. Nevertheless the results compare favorably with the scarce data about the application of PRF with the traditional monopolar or bipolar by two needle approaches in PSPS type 2 patients. We postulate that the introduction of the OCF electrode configuration could made the difference. The voltage in PRF literature has always been expressed in volts (28).

We estimate that in this study the distance between the cathode and the anode was no more than 3–4 cm. Since the applied voltage was 25 V, the electric fields in the largest part of the spheroid must have been approximately 200–400 V/m. These values are concordant with the fields that are generated by transcutaneous PRF, a method that has been proven to be effective in a RCT study (29). The results of this study are concordant with the concept that the low range of electric fields initiate the effects of PRF.

On this technique, the principal item to consider is the density of current in the pathologic area to treat. The relation that determine the density of electrostatic charge in the pathologic area is:


J=DeltaV/RxA

where: J = density of electrostatic charge; Delta V = Voltage; R = Resistence; A = distribution area of charge.

If we consider the same resistance in the two cases (monopolar system with ground plate and OCF), the two variables are the voltage and the distribution area of the charge.

In this case, the ratio between the surface area of the needle and ground plate is much bigger than the difference of voltage in the two systems. The result is the increase in the density of the allostatic charge in the pathologic small area with OCF, also with the same electric field value and resistance as the conventional monopolar system. The increase in the electrostatic charge could explain the better results despite the same electric field value, and this could improve the clinical results and at the same time reduce the risk of iatrogenic damage of the nervous tissue.

The results of PRF in PSPS type 2 performed with an electrode placed through the sacral hiatus was reported to be successful against pain in 32% of patients (21); the electrodiagnostic findings of neuropathy indicate a lower success rate compared with patients suffering from radiculopathy or with normal findings (30) with a 26%–35% good results at 12 months follow-up. Nevertheless we believe that PRF with the addition of OCF has some advantages over other methods.

Secondly, with this new electrode configuration there is no need to enter the inflamed or scar area. RF current easily crosses barriers such as scar tissue or other tissue barriers, leaving the current density grossly intact as long as dilution of the current for geometrical reasons does not take place. Using the OCF bipolar system effectively reduces this “geometrical dilution” (Figure 1) of the current. An electrode position just outside the inflamed area is therefore sufficient, and this is thought to contribute to the safety of the method. Moreover different position of the needle at the level of the ganglion do not affect the clinical result (31). In case of treatment of multiple ganglia as is often required in cases of PSPS type 2, it is sufficient to direct the tip of the lead towards the overlying ganglion and use only one needle as the cathode for ganglion. With the steerable tip you can get as close as possible to the ganglion or the scar that surrounds it.

We believe that PRF is an innovative method that has already demonstrated good results in other types of painful pathologies so: it is time to develop an effective technique also for PSPS.

This new modality of PRF in patients with PSPS type 2 seems not to be inferior to procedures done with a needle, for patients with radiculopathy without previous spinal surgery (32). Further prospective double-blind randomized long-term studies with a significant number of patients are required for the validation of this technique. Our clinical sample is too small to demonstrate the definitive effectiveness of this technique so further studies are needed to elucidate whether this procedure requires only one neddle (cathode) even if multiple ganglia are targeted. We believe that the minimum follow-up of PRF in PSPS type 2 should be at least 12 months, and studies should also specify how many levels are involved and whether there is bilaterality.

## Data availability statement

The raw data supporting the conclusions of this article will be made available by the authors, without undue reservation.

## Ethics statement

The studies involving humans were approved by the Medical Management Direction of ASST Settelaghi (Varese, Italy). The studies were conducted in accordance with the local legislation and institutional requirements. The participants provided their written informed consent to participate in this study. Written informed consent was obtained from the individual(s) for the publication of any potentially identifiable images or data included in this article.

## Author contributions

AD and SC contributed to the clinical data collection and literature review. All authors contributed to the article and approved the submitted version.

## Footnotes

^1^ https://www.socscistatistics.com/tests/anova/default2.aspx
